# New Contributions to *Pseudonapomyza* (Diptera: Agromyzidae) from Spain: Addition of Three New Species

**DOI:** 10.1673/031.010.14129

**Published:** 2010-10-04

**Authors:** Ricardo Gil-Ortiz, Michel Martinez, Ricardo Jiménez-Peydró

**Affiliations:** ^1^ Laboratorio de Entomología y Control de Plagas, Instituto Cavanilles de Biodiversidad y Biología Evolutiva, Universität de Valencia, Apartado Oficial 22085, 46071 Valencia, SPAIN; ^2^ Present address: INRA, Centre de Biologie pour la Gestion des Populations (CBGP), Campus International de Baillarguet - CS 30 016, 34988 Montferrier-sur-Lez cedex, France

**Keywords:** *Pseudonapomyza curvata* n. sp., *Pseudonapomyza longitata* n. sp., *Pseudonapomyza sicicornis* n. sp

## Abstract

The genus *Pseudonapomyza* (Diptera: Agromyzidae) includes the main leafminer pests for monocots. Three new species are described that were captured using Malaise traps in “Tinença de Benifassà”, “Font Roja” and “Lagunas de La Mata-Torrevieja” (Spain) Natural Parks: *Pseudonapomyza curvata* n. sp., *P. longitata* n. sp., and *P. sicicornis* n. sp. Systematics. Ecological data are discussed.

## Introduction

*Pseudonapomyza* genus belongs to the subfamily Phytomyzinae within the family Agromyzidae (Diptera). In temperate zones of Northern and Southern hemispheres *Pseudonapomyza* mines leaves exclusively on monocots (Gramineae family) (Spencer 1990).

So far only 9 species in the *Pseudonapomyza* genus have been cited in Spain *Pseudonapomyza atra* (Meigen, 1830); *P. hispinica* Spencer, 1973; *P. insularis* Zlobin, 1993; *P. lacteipennis* (Malloch, 1913); *P. spinosa* Spencer, 1973; *P. strobliana* Spencer, 1973; *P. vota* Spencer, 1973 (listed by Martinez and Báez 2002); *P. europaea* Spencer, 1973 (cited by Cerny 2004); and *P. mediterranea* Gil-Ortiz, 2010 (in press). Overall 92 species are spread across most of the geographical regions except South America (Zlobin, 2002). Forty-eight species at the level of the Palaearctic region are known, including the species described in this manuscript.

The important pest species of this genus are *Pseudonapomyza asiatica* Spencer, 1961 (Liao and Shiao 2001) in Taiwan, *P. gujaratica* Shah, 1982 in India or *P. spicata* (Malloch, 1914) in the Philippines (Litsinger and Barrion 1987). Benavent-Corai (2004) cites *P. atra* (Meigen, 1830) and *P. spinosa* Spencer, 1973 as species of economic interest in Spain. Cereals with the most agronomic importance susceptible to be attacked by members of the *Pseudonapomyza* genus in Spain are in the genera *Avena, Secale* and *Triticum* (Benavent-Corai et al. 2005).

Normally, low populations of these species are controlled naturally by parasitoids. The misuse of pesticides and other human actions can break this balance or cause the development of pest species that previously were not a concern. Getting to know the overall biodiversity of Agromyzidae is a preventive tool for present and future pest control.

**Figure 1.**
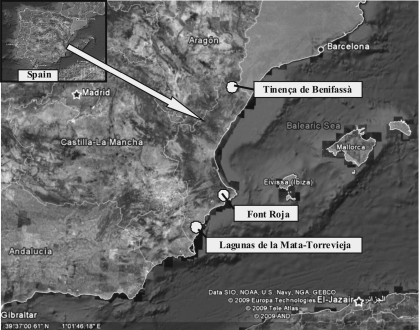
Location of the areas studied in the Valencian Community. High quality figures are available online.

**Studied areas** (Figure 1). *Tinençde Benifassà Natural Park* (Castellón): it is located in the Northern part of the Valencian Community bordering the Tarragona and Teruel provinces. The surface area of the park is around 25.8 hectares, with minimal anthropological impact (<250 residents). It presents high faunistic and vegetal biodiversity including well preserved woodlands of pine and oak, scrubland composed of typical mediterranean vegetation including a high number of endemisms and crop areas. It typically snows in the winter (5ndash;50 mm) and average temperatures are around 9–22° C in winter and summer, respectively. Annual rainfall is around 450–550 mm.

*Font Roja Natural Park* (Alicante): it is located in the Alicante province. It is basically a holm oak mountain composed of Tertiary calcareous rocks. The biodiversity of vegetation is high, including different areas composed of deciduous wood, shady evergreen or holm oak groves, sunny brushwood zones, rock vegetation, rubble vegetation, pine woods and crops. Annual rainfall is comprised around 350–450 mm, and average temperatures are around 7–23° C in winter and summer, respectively.

*Lagunas de la Mata-Torrevieja Natural Park* (Alicante): it is located in the southern point of Valencian Community. It is characterized by saline soils, semiarid climate, annual precipitations lower than 300 mm, and average temperatures around 13–27° C in winter and summer, respectively. There are salt marsh areas, carrizal-juncal zones and scrubland. Fresh vegetation is present until mid-May, later the high temperatures (>35° C) destroy practically all annual plants.

**Capture system.** Insects were captured using one Malaise trap per site (Model G700, Entomopraxis-Barcelona-Spain), that proved to be highly effective in monitoring the evolution of populations of Agromyzidae flies (von-Tschirnhaus 1992) throughout the years 2004 to 2006. Captures were collected weekly, except that the traps were removed when it snowed. 70 vol. alcohol was used for specimen conservation. The GPS coordinates of Malaise traps placement were 40°39′22.6″/E00°09′26.8″ (755 m altitude) at *Tinença de Benifassà*, 38°39′43.1″/W00°31′ 04.0″ (1076 m) at *Font Roja*, and 38°01′48.8″/W00°42′00.1″ (5 m) at *Lagunas de La Mata-Torrevieja.*

**Diagnosis.** The presence of a subcostal extremely short vein compared to the rest of Agromyzidae specimens, as well as the characteristic pointed antenna (Zlobin, 2002) were the main diagnostic characters used to separate specimens of the genus *Pseudonapomyza.* Identifications were exclusively carried out with male specimens. This study mainly uses the Diptera terminology system proposed in the Manual of Nearctic Diptera by McAlpine (1981). The dorsocentral *(dc)* bristles are numbered from posterior to anterior on the thorax.

## Results and Discussion

Below three new species for science are presented: *Pseudonapomyza curvata* n. sp., *P. longitata* n. sp., and *P. sicicornis* n. sp.

### *Pseudonapomyza curvata* Gil-Ortiz n. sp.

**Holotype male:** Castellón (Spain). *Tinença de Benifassà:* Collected from 20–28 August 2006. Specimens were Deposited in the Entomological Collection of Universidad de Valencia (ENV).

**Paratypes:** Same locality and position of holotype, 2 ♂, 1 – 10 August 2006; Alicante (Spain). Font Roja: 1 ♂ 24 June 2004 – 1 July 2004; 2 ♂, 29 July 2004 – 2 August 2004; 1 ♂, 2 – 9 August 2004; 1 ♂ 9 – 16 August 2004; 1 ♂, 30 August 2004 – 6 September 2004; 1 ♂, 6–13 September 2004; 1 ♂, 4 – 11 October 2004. All paratypes were deposited in ENV.

**Derivatio nominis.** This new species is named according to the particular aedeagus shape.

**Figure 2.**
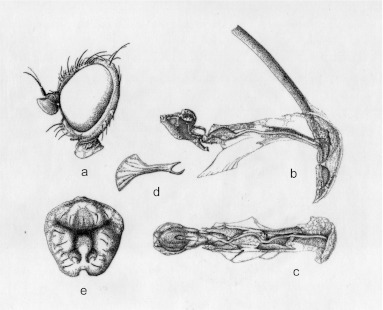
*Pseudonapomyza curvata* n. sp. Holotype ♂: a- Head in lateral view; b- aedeagus in lateral view; c- aedeagus in ventral view; d- sperm pump in lateral view; e- epandrium in anterior view. High quality figures are available online.

**Description. Head** (Figure 2a). Frons only clearly prominent at level of lunule. 3^rd^ antennal segment pointed at upper corner, as long as wide, minutely pubescent with short brown pilosity uniformly distributed. Arista normal, with very fine and very short pilosity. Fronto-orbital plate (= parafrontalia) with 2 *ors* (upper orbital) and 3 curved inwards *ori* (lower orbital). Orbital setulae short (9–10) and reclinated. Ocellar triangle 0.12 × 0.11 mm slightly longer than wide, extends to level of upper *ors.* Two ocellar bristles (*oc*) a little divergent, slightly smaller and as strong as *ors.* Two postocellar bristles (*poc*) clearly divergent and a little longer than *oc.* Internal bristle (*vti*) (= inner vertical setae [*i vt s*]) long and strong, much longer than *ors* and *ori.* External vertical bristle (*vte*) (= outer vertical setae [*o vt s*]) strong but much smaller than *vti* (on average, *vti* 1.5 times longer than *vte).* Inter-ocular space measured (in frontal view) at level of *ors* = 0.9 X eye (in profile, at a highest measurement). Cheeks forms *arc* below eye. Gena including cheeks (at highest measurement) = 0.28 X eyes (in profile at highest measurement). Eyes without pilosity.

**Thorax.** Mesonotum with 3+0 long and strong dorsocentral bristles (*dc*) increasing in size to scutellum. *acr* numerous (9–10) irregularly arranged in 5–6 no spaced rows. Intra alar seta (*ia*) small, about same size as *acr.* Anterior and posterior supra alar setae (*spal*) as long and strong as first and second *dc.* Humeral cali with 1 anterior bristle accompanied by 4–5 small setulae. Notopleura with 2 normal notopleural bristles. Posterior part of anapisternum (mesopleura) with 1 strong bristle, and generally 1 small setula at each side. Katepisternum (sternopleura) with 1 strong bristle situated at supero-posterior angle. Disc of scutellum without particular seta except usual 4. 2 apical scutellar setae (*ap sctl s*) generally parallel or very slightly convergent; 2 basal scutellar seta (*b sctl s*) about same size as *ap sctl s*, parallel or slightly directed outwards. Wing: length (on average) 1.5 × 0.63 (long × wide) mm. Thickening of costal (*C*) vein, clearly reaching *R4+5* ending much before wing tip. Second and third costal section short. In proportion the length from first to fourth costal section is approximately 1:0.63:0.45:0.85. Discal cell (*dm*) and transverse (*dm-cu*) [second crossvein] missing. Legs: with normal pilosity with the usual pre-apical bristle.

**Abdomen.** Setae of the tergites very distinct and relatively numerous arranged stronger on the posterior marginal border.

**Coloration.** Head entirely brownish. Face, front and orbital stripes brown. Lunule light brown. Inner vertical setae (i *vt s = vti*) and outer vertical setae *(0 vt s = vte)* on brown ground. Ocellar triangle dark brown like cheeks. Gena light brown. Torax and scutellum uniformly brown. Calypteral fringe light brown close to wing. Halter whitetransparent. Legs entirely brown. Abdomen brown on the upper side and light brown on the bottom side. Tergites 1 to 5 with a clear darker brown band between contiguous margin, with wide bottom brownish spots.

**Aedeagus and associated structures.** Aedeagus (Figures 2b and 2c). Cercus short and thin. Sperm pump (= ejaculatory apodeme) longer (0.2 mm) than wide (0.12 mm) (wider part), different from the other two species described (Figure 2d). Surstylus (= gonostylus) with dense pilosity inside of each lower corner (Figure 2e). In ventral view the distiphallus presents a typical round shape while in lateral view 2 curved structures are present.

**Bionomy.** Unknown host-plants.

**Figure 3.**
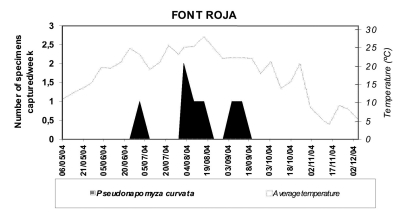
Space-time evolution of *Pseudonapomyza curvata* n. sp. captured in “Tinença de Benifassà” Natural Park. High quality figures are available online.

**Phenology** (Figure 3). In “Font Roja” this species was found from late June to mid October with average temperatures of 22.4 – 28.2^°^C (36.4° C max. and 17.1° C min.). In 2004 three generations were produced but low captures made it difficult to exactly predict the development of this species. Captures of “Tinença de Benifassà” were produced in August with average temperatures of 23.5 – 24° C (31° C max. and 17° C min.).

**Systematic position.** This species is well characterized by the particular shape of the aedeagus. This new species is closely related to *Pseudonapomyza atra* (Meigen, 1830). This last species has been found undermining the genus *Apera, Avena, Hordeum, Lolium, Phalaris, Poa, Secale, Triticum* (Spencer, 1990), and *Holcus* (Parkman et al. 1989). The fundamental difference between these two species is the distinct curvature of distiphallus which is closest in the case of *P. atra.*

### *Pseudonapomyza longitata* Gil-Ortiz n. sp.

**Holotype male:** Castellón (Spain). Tinença de Benifassà: Collected from 6–13 June 2005, and deposited in ENV.

**Paratypes:** Alicante (Spain). Font Roja: 1 ♂, 17 – 24 June 2004; 15 ♂ 24 June 2004 – 1 July 2004; 6 ♂, 1 – 8 July 2004; 3 ♂ 15 – 22 July 2004; 2 ♂, 22 – 29 July 2004; 7 ♂, 29 July 2004 – 2 August 2004; 6 ♂, 2 – 9 August 2004; 7 ♂, 9 – 16 August 2004; 6 ♂ 16 – 23 August 2004; 4 ♂, 23 – 30 August 2004; 1 ♂, 30 August 2004 – 6 September 2004; 1 ♂, 23 – 30 May 2005; 1 ♂, 13 – 20 June 2005; 1 ♂, 27 June 2005 – 4 July 2005; 6 ♂, 11 – 18 July 2005; 1 ♂, 1 – 8 August 2005; 1 ♂, 15 – 22 August 2005; 1 ♂, 22 – 29 August 2005; 1 ♂, 4–11 May 2006; 1 ♂, 29 May 2006 – 5 June 52006; 1 ♂, 5 – 12 June .vi.2006; 2 ♂, 12 – 19 June 2006; 9 ♂, 19–26 June; 2006; 4 ♂ 26 June 2006 – 3 July 2006; 3 ♂, 3 – 10 July 2006; 1 ♂, 17 – 25 July 2006; 6 ♂, 25 – 31 July 2006; 1 ♂, 31 July 2006 – 7 August 2006; 3 ♂, 7 – 14 August 2006; 2 ♂ 14 – 21 August 2006; 2 ♂, 21 – 28 August 2006 and 1 ♂, 4 – 11 September 2006. Lagunas de La Mata-Torrevieja: 1 ♂, 9 – 16 August 2005; 1♂, 6 – 13 December 2005; 1 ♂, 21 – 28 March 2006; 1 ♂, 31 October 2006 – 7 November 2006; 1 ♂, 20 February 2007 – 6 March 2007; 1 ♂, 6 – 13 March 2007 and 1 ♂, 13 – 20 March2007. All paratypes were deposited in ENV.

**Derivatio nominis.** This new species is named according to the aedeagus shape.

**Figure 4.**
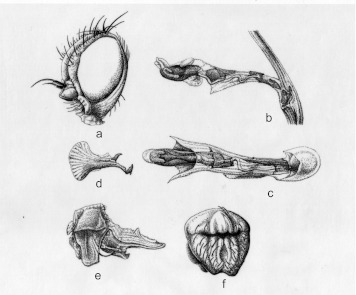
*Pseudonapomyza longitata* n. sp. Holotype ♂: a- Head in lateral view; b- aedeagus in lateral view; c- aedeagus in ventral view; d- sperm pump in lateral view; e- epandrium in lateral view; f- epandrium in anterior view. High quality figures are available online.

**Description.** As *P. curvata*, except: **Head** (Figure 4a). Frons moderately prominent between eyes in profile (more pronounced at the height of the lunule). 3^rd^ antennal segment strongly pointed at upper corner, as long as wide, minutely pubescent with short brown pilosity, these clearly more distinct on the border of the antenna. Fronto-orbital plate (= parafrontalia) with 1 *ors* (upper orbital). Orbital setulae short (minimum 12) erected along *ori* and reclinated along *ors* in an only row. Ocellar triangle longer than wide 0.13 × 0.11 mm. Two ocellar bristles (*oc*) slightly divergent or parallel. Two postocellar bristles (*poc*) slightly divergent and equal or slightly longer than *oc.* Inter-ocular space measured (in frontal view) at level of *ors* = 1.7 X eye (in profile, at a highest measurement). Gena including cheeks (at highest measurement) = 0.32 X eyes (in profile at highest measurement).

**Thorax.** *acr* numerous (10–12) irregularly arranged in 8 no spaced rows. Wing: length (on average) 1.4 × 0.6 (long × wide) mm. In proportion the length from first to fourth costal section is approximately 1:0.7:0.2:0.7.

**Abdomen.** Setae of the tergites very distinct and relatively numerous arranged on dorsal part, while on ventral side fine pilosity is present.

**Coloration.** Lunule dark brown.

**Aedeagus and associated structures.** Aedeagus (Figures 4b and 4c). Sperm pump (= ejaculatory apodeme) longer (0.18 mm) than wide (0.1 mm) (wider part) expanded uniformly on the two sides (Figure 4d). Surstylus (= gonostylus) (Figures 4e and 4f).

**Bionomy.** Unknown host-plants.

**Figure 5.**
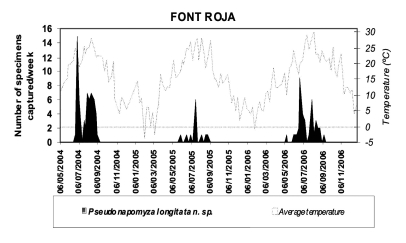
Space-time evolution of *Pseudonapomyza longitata* n. sp. captured in “Font Roja” Natural Park. High quality figures are available online.

**Phenology** (Figure 5). According to the captures produced in “Font Roja”, 3–6 generations were distributed from mid-May to mid September. The largest captures were 15 males per week in late June with an average temperature of 23.2° C (28.9° C max. and 17.5° C min.). The capture in “Tinença de Benifassà” was made in early/mid June with average temperatures of 19° C (23° C max. and 15° C min.). Captures of this species in “Lagunas de La Mata-Torrevieja” were low and occurred irregularly, being in the range of average temperatures of 12.5–27° C (28° C max. and 8° C min.).

**Systematic position.** Compared with the rest of the Palaearctic species, higher affinity toward *Pseudonapomyza europaea* Spencer, 1973 is observed, but it differs in the shape of distiphallus being shorter and more rounded in the case of *P. longitata.*

**Figure 6.**
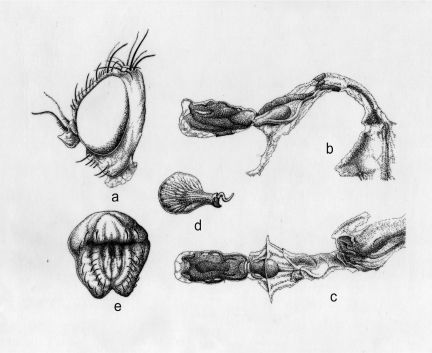
*Pseudonapomyza sicicornis* n. sp. Holotype ♂: a- Head in lateral view; b- aedeagus in lateral view; c- aedeagus in ventral view; d- sperm pump in lateral view; e- epandrium in anterior view. High quality figures are available online.

### *Pseudonapomyza sicicornis* Gil-Ortiz n. sp.

**Holotype male:** Spain: Castellón. *Tinença de Benifassà:* Collected from 20 – 28 August 2006, deposited in ENV. **Paratype:** neither.

**Description.** As *P. curvata*, except, **Head** (Figure 6a). 3^rd^ antennal segment strongly pointed at upper corner, as long as wide, minutely pubescent with short brown pilosity, these clearly more distinct in the body of the antenna. Fronto-orbital plate (= parafrontalia) with 2 curved inwards *ori* (lower orbital). Orbital setulae short (10–12) slightly reclinated along *ori* and reclinated along *ors* in an only row. Ocellar triangle as long as wide 0.1 × 0.1 mm. Two ocellar bristles (*oc*) slightly divergent or parallel. Two postocellar bristles (*poc*) slightly divergent and equal or slightly longer than *oc.* Inter-ocular space measured (in frontal view) at level of *ors =* 1.1 X eye (in profile, at a highest measurement). Gena including cheeks (at highest measurement) = 0.25 X eyes (in profile at highest measurement).

**Thorax,** *acr* numerous (minimum 10) irregularly arranged in 8–9 no spaced rows. 2 basal scutellar setulae (*b sctl s*) slightly directed inwards. Wing: length (on average) 1.5 × 0.65 (long × wide) mm. In proportion the length from first to fourth costal section is approximately 1:1:0.4:1.

**Aedeagus and associated structures.** Aedeagus (Figures 6b and 6c). Sperm pump (= ejaculatory apodeme) longer (0.22 mm) than wide (0.14 mm) (wider part) expanded uniformly on the two sides (Figure 6d). Surstylus (= gonostylus) with slight pilosity inside of each lower corner (Figure 6e).

**Bionomy.** Unknown host-plants.

**Phenology.** This species has been captured when average temperatures were 23.5° C (19° C min. and 28° C max.). Based on the captures an only generation in summer is observed, being difficult to predict the evolution of this species. Although most probable that it is also present in summer and autumn with several generations.

**Systematic position.** This species is characterized by having a particular morphology of the aedeagus. Comparing the aedeagus morphology with the rest of Palaearctic species the closest species are *Pseudonapomyza siciformis* Zlobin, 2002 and *P. strobliana* Spencer, 1973. The biggest differences in distiphallus of both cases is the particular curvature of the upper horn and the bottom round shape.
